# Supplementary material for: Nuances in intensity deviant asymmetric responses as a biomarker for tinnitus
Source: PLoS One. 2023 Aug 7;18(8):e0289062. doi: 10.1371/journal.pone.0289062 (PMC10406247; doi:10.1371/journal.pone.0289062)
Supplement: S1 Table — (DOCX) [file pone.0289062.s001.docx]

| Edge | 5.31E+03 | 4.24E+03 | 7.22E+03 | 2.90E+03 | 7.97E+03 | 6.73E+03 | 2.39E+03 | 3.25E+03 | 4.71E+03 | 5.65E+03 | 3.04E+03 | 6.36E+03 | 6.37E+03 | 4.93E+03 |
| --- | --- | --- | --- | --- | --- | --- | --- | --- | --- | --- | --- | --- | --- | --- |
| Centre | 4.52E+03 | 2.64E+03 | 6.03E+03 | 3.21E+03 | 6.58E+03 | 4.88E+03 | 2.51E+03 | 3.25E+03 | 4.21E+03 | 6.06E+03 | 4.11E+03 | 5.36E+03 | 8.19E+03 | 4.88E+03 |
