# Supplementary material for: Nuances in intensity deviant asymmetric responses as a biomarker for tinnitus
Source: PLoS One. 2023 Aug 7;18(8):e0289062. doi: 10.1371/journal.pone.0289062 (PMC10406247; doi:10.1371/journal.pone.0289062)
Supplement: Attachment — Submitted filename: Response to Reviewers.docx [file pone.0289062.s003.docx]

Dear editors,

Thank you for reviewing our manuscript and providing constructive comments. I am very grateful for your time and suggestions. In this letter, I aim to address the issues brought up by the journal and the reviewers.

Journal requirements:

1) “Funding information should not appear in the Acknowledgments section or other areas of your manuscript.”

I have removed the funding section from the main manuscript file (line 493). I also included a funding statement in the cover letter.

2) “Please review your reference list to ensure that it is complete and correct.”

I believe that my reference list is complete and does not include any retracted papers.

Responses to reviewer 1:

1) “Sampling size can be increased for future investigation purposes.”

Thank you for your comment! While the sample size could have been larger, we believe that it should have been sufficient to attempt replication of previous findings. I have added a statement about this to the manuscript (line 106): “The sample size was small largely because this study was seeking the kind of large and invariant effects indicated in the previous study”.

Responses to reviewer 2:

1) “Justification of sample size”

While the sample size could have been larger, we believe that it should have been sufficient to attempt replication of previous findings. I have added a statement about this to the manuscript (line 106): “The sample size was small largely because this study was seeking the kind of large and invariant effects indicated in the previous study”.

2) “Specify the age range of participant (both control and clinical group)”

I have now put this information in Table 1 (line 226; tinnitus group range 20-80 and control group range 22-70)

3) “What is the starting level of intensity of band pass noise in psychophysical assessment?”

I added this (line 134-136): “The intensity was based on an inverse Fourier transform of a Hanning spectrum noise, with peak amplitude at the centre frequency equal to 1. It was not a specific dB value, and it was always a relatively quiet stimulus which subjects needed to increase in intensity for the match.”

4) “How the psychophysical assessment performed in bilateral tinnitus.”

I have added this on line 133: “In cases of bilateral tinnitus, the participants heard the sound in both ears, and could adjust the ear balance.”

5) “Provide the details of tinnitus pitch, center frequency of tinnitus of each participant.”

I have now put this in as a supplemental table.

6) “What is the duration of standard stimulus?”

300 ms (line 171)

7) “The experimental design closely followed the paradigm used (1), with the exception of using a 1 kHz tone as the control condition - consider to rephrase”

I have now changed this to “The experimental design closely followed the paradigm used in the original study (1), with the additional inclusion of a 1 kHz tone as a control condition.” (line 167)

8) “The tones were presented isochronously to the ear(s) that the tinnitus participant indicated as the tinnitus ear(s), or the same ear(s) for their matched control (illustrate it with suitable example).”

I added “For example, if the participant with tinnitus only had tinnitus in their right ear, the matched control would also only hear the tones in their right ear.” (line 174).

9) “In Method - ‘with chronic tinnitus for over 6 months’ and in results according to the THI, tinnitus was causing a slight problem to four participants, mild problem to seven participants, a moderate problem to two participants and a severe problem to one participant. - Conflicting statement and THI representation (27.29 (16.69)*) in Table-2.”

This seems consistent to me; the largest frequency of participants scored between 18 and 36 (mild problem), which is reflected by the mean score being within this category also. I have included the ranges of category scores in the description (line 212): “According to the THI, tinnitus was causing a slight problem to four participants (<16 THI score), a mild problem to seven participants (18-36), a moderate problem to two participants (38-56) and a severe problem to one participant (58-76). Tinnitus duration ranged from 4 to 60 years. The duration was skewed to the left, so the mean (21) was lesser than could be expected in a normal distribution.”

10) “Tinnitus duration ranged from 4 to 60 years and in table-2 the duration in years 21 (19.16) - check it.”

This is also accurate based on my data. I have included an explanation for this (line 214): “the duration was skewed to the left, so the mean is lesser than could be expected in a normal distribution”.

11) Mention the tinnitus frequency and edge frequency for each participant in a table.

This is now in the supplemental table based on comment 5).

12) “There is a mismatch between Participant age, hearing loss pattern and tinnitus duration range. Do the participants in control groups are older adults, in audiogram it is observed that > 4 kHz above >25 >30 dB HL (Figure-1).”

The figure is representative of the PTA results I saw, with many participants starting to have some hearing loss from 6 kHz. The largest tinnitus duration occurred in one of my oldest participants, who indicated that they developed tinnitus as a teenager. The age and hearing loss means, and overall distributions were not significantly different between groups (line 233).

13) Specify the ocular channel used in recording the EEG data.

I have now included this (line 164): “No ocular channel was used as the standard EEG channels were sufficient for removing ocular artifacts”.

14) The mean dB difference between the loud and quiet stimuli was not significant between groups, however (Man U test, p =0.376) - Rephrase the sentence.

Changed this to “Nonetheless, the mean dB difference between the loud and quiet stimuli was not significant between the tinnitus and control groups (Man U test, p =0.376).” (Line 250).

15) Possible reasons for the findings are missing under each heading of the discussion section.

Thank you for the good suggestion. Upon further consideration, given that my discussion subsections are quite short, I am not sure that a summary at the beginning of each section would be needed, and I would prefer to avoid repetition.

Thank you to the journal for considering this paper and to the reviewers for their comments! I hope this response is satisfactory to any concerns raised.

Kind regards,

Ekaterina Yukhnovich
